# Characterization of Histone H2A Derived Antimicrobial Peptides, Harriottins, from Sicklefin Chimaera *Neoharriotta pinnata* (Schnakenbeck, 1931) and Its Evolutionary Divergence with respect to CO1 and Histone H2A

**DOI:** 10.1155/2013/930216

**Published:** 2013-06-02

**Authors:** Naveen Sathyan, Rosamma Philip, E. R. Chaithanya, P. R. Anil Kumar, V. N. Sanjeevan, I. S. Bright Singh

**Affiliations:** ^1^Department of Marine Biology, Microbiology and Biochemistry, School of Marine Sciences, Cochin University of Science and Technology, Fine Arts Avenue, Kochi 682016, Kerala, India; ^2^Centre for Marine Living Resources and Ecology, Kakkanad, Kochi 682037, Kerala, India; ^3^National Centre for Aquatic Animal Health (NCAAH), CUSAT, Fine Arts Avenue, Kochi 682016, Kerala, India

## Abstract

Antimicrobial peptides (AMPs) are humoral innate immune components of fishes that provide protection against pathogenic infections. Histone derived antimicrobial peptides are reported to actively participate in the immune defenses of fishes. Present study deals with identification of putative antimicrobial sequences from the histone H2A of sicklefin chimaera, *Neoharriotta pinnata*. A 52 amino acid residue termed Harriottin-1, a 40 amino acid Harriottin-2, and a 21 mer Harriottin-3 were identified to possess antimicrobial sequence motif. Physicochemical properties and molecular structure of Harriottins are in agreement with the characteristic features of antimicrobial peptides, indicating its potential role in innate immunity of sicklefin chimaera. The histone H2A sequence of sicklefin chimera was found to differ from previously reported histone H2A sequences. Phylogenetic analysis based on histone H2A and cytochrome oxidase subunit-1 (CO1) gene revealed *N. pinnata* to occupy an intermediate position with respect to invertebrates and vertebrates.

## 1. Introduction

Antimicrobial peptides (AMPs) are ubiquitous and multipotent components of humoral innate immune response of most living organisms against invasion by pathogens [1]. The characteristics of naturally occurring AMPs, such as relatively small size (12–50 amino acids), cationicity, and amphipathicity allow them to interact with and penetrate into the membranes by the formation of transmembrane ion permeable pores or by a detergent-like manner, resulting in the leakage of the cytoplasmic components and cell death [2]. In the last two decades a considerable number of gene coded AMPs, either inducible or constitutive, with broad spectrum activity against different types of pathogens, have been reported from wide range of organisms, and their significance in innate immunity is becoming more and more appreciated. The specific immune mechanisms in the primeval vertebrates such as fish are less developed than those of higher vertebrates [3, 4] and are limited by temperature restraints on their metabolism [5]. Therefore, fish rely highly on their innate immune mechanisms for protection against invading pathogens and this makes them a potential candidate for antimicrobial peptide research. 

Histone derived antimicrobial peptides form an important category of AMPs and is reported from a number of vertebrates and invertebrates [6]. N-terminus of histone H2A is rich in basic amino acids, a characteristic which allows histone H2A to act as a precursor for antimicrobial peptides [7]. In case of marine fishes AMPs derived from the N-terminus domain of histone H2A have been reported from a number of species including catfish *Parasilurus asotus* [8], Atlantic salmon *Salmo salar* [9], Atlantic halibut *Hippoglossus hippoglossus* [10], rainbow trout *Oncorhynchus mykiss *[11], round whip ray *Himantura pastinacoides *[12], and recently from two marine teleost fishes, *Tachysurus jella* and *Cynoglossus semifasciatus* [13]. Histone derived AMPs have also been reported from marine invertebrates including Pacific white shrimp *Litopenaeus vannamei* [14], scallop *Chlamys farreri* [7], abalone *Haliotis discus*, Marine Clam *Sunetta scripta* [15], and from few other marine molluscs [16].

The role of antimicrobial peptides in the innate immune response of fishes belonging to the family Holocephali has not been yet studied in detail. The present study was carried out to get a deeper insight into the role of histone H2A derived AMPs in the immune response of sicklefin chimaera *Neoharriotta pinnata*. Here we report the identification and characterization of antimicrobial peptide sequences derived from histone H2A of *N. pinnata.* This is the first report of histone derived antimicrobial peptides from Holocephali group of fishes. The paper also describes in detail the divergence in molecular evolution of histone H2A in *N. pinnata *and related fishes. Also the evolutionary relationship of Holocephalan fishes to other organisms based on the nucleotide sequence of cytochrome oxidase subunit I have been discussed in detail.

## 2. Methodology

### 2.1. Sample Collection

Live *N. pinnata* was caught from a depth of 500 m off Karaikkal Coast, Tamil Nadu, India, during Cruise number 291 of Fisheries and Oceanography Research Vessel *Sagar Sampada* (Ministry of Earth Sciences, Government of India). High Speed Demersal Trawl (HSDT) net operated on-board was employed for capturing the species. Blood was collected from the lamellar artery near gill region using specially designed capillary tubes (RNase free) and rinsed in precooled anticoagulant solution (RNase free 10% sodium citrate, pH 7). Blood was homogenized in TRI reagent (Sigma) and stored at −20°C on-board in the Biological Laboratory facility of the research vessel. 

### 2.2. RNA Isolation and cDNA Synthesis

Total RNA was isolated from blood cells using TRI reagent (Sigma) and following manufacturer's instructions. Purity and quality of RNA were checked on 0.8% agarose gel. First strand cDNA was generated in a 20 *μ*L reaction volume containing 5 *μ*g total RNA, 1x RT buffer, 2 mM dNTP, 2 mM oligo d(T20), 20 U of RNase inhibitor, and 100 U of MMLV reverse transcriptase (New England Biolabs, USA). The reaction was conducted at 42°C for 1 h followed by an inactivation step at 85°C for 15 min. Gene-specific primers forward (5′-GATCATGTTCGAGACCTTCAACAC-3′) and reverse (5′-CGATGGTGATGACCTGTCCGTC-3′) were used to amplify a product of 389 bp constitutive expression gene, the beta-actin as an internal control to verify the RT-PCR reaction.

### 2.3. PCR Amplification

Amplification of histone H2A derived antimicrobial peptide sequence from cDNA of *N. pinnata* was done using forward primer (5′-ATGTCCGGRMGMGGSAARAC-3′) and reverse primer (5′-GGGATGATGCGMGTCTTCTTGTT-3′) [10]. PCR amplification of 1 *μ*L of cDNA was performed in a 25 *μ*L reaction volume containing 1x standard Taq buffer (10 mM Tris-HCl, 50 mM KCl, pH 8.3), 1.5 mM MgCl_2_, 200 mM dNTPs, 0.4 mM each primer, and 1 U Taq DNA polymerase (New England Biolabs). The thermal profile used was an initial denaturation at 94°C for 2 minutes followed by 35 cycles of 94°C for 15 seconds, 60°C for 30 seconds, and 68°C for 30 seconds and a final extension at 68°C for 10 minutes. PCR products were analyzed by electrophoresis in 1.5% agarose gel in TBE buffer, stained with SYBR Safe and visualized under UV light. 

### 2.4. TA Cloning and Sequencing

The purified PCR products were ligated into the pTZ57R/T easy clone vector and transformed using competent *E. coli* cells, JM107 as per manufacturer's protocols (InsTAclone PCR Cloning Kit, Fermentas). Transformed bacteria were cultured in Luria Bertaini agar plates containing ampicillin, IPTG, and X-gal at 37°C for 24 h, and the recombinant clones with the inserts were selected by blue white screening. The white colonies were selected and streaked on to fresh ampicillin plates and screened using vector specific primers, M13 F (5′-GTAAAACGACGGCCAG-3′) and M13 R (5′-CAGGAAACAGCTAT GAC-3′) and histone H2A sequence specific primers. For M13 primers the thermal profile used was 94°C for 5 minutes followed by 35 cycles of 94°C for 30 seconds, 54°C for 30 seconds, and 72°C for 30 seconds and a final extension at 72°C for 10 minutes. Amplicons obtained were sequenced using ABI Prism BigDye Terminator Cycle Sequencing Ready Reaction kit on an ABI Prism 377 DNA sequencer (Applied Biosystem) at SciGenom Sequencing Facility, India.

### 2.5. Taxonomic Identification

For taxonomic identification of the species genomic DNA was isolated using TRI reagent (Sigma). The concentration of isolated DNA was estimated using a UV spectrophotometer (Hitachi U-2900). The DNA was diluted to a final concentration of 100 ng/*μ*L. The cytochrome oxidase-I (CO1) gene was amplified in a 25 *μ*L reaction volume containing the above said PCR reagents in same concentration. 1 *μ*L of genomic DNA was used as template. The primers used for the amplification of CO1 gene were LCO1490 (5′-GGTCAACAAATCATAAAGATATTGG-3′) and HC02198 (5′-TAAACTTCAGGGTGACCAAAAAATCA-3′) [17]. The thermal regime consisted of an initial denaturation at 95°C for 5 minutes followed by 35 cycles of 95°C for 45 seconds, 50°C for 30 seconds, and 72°C for 45 seconds and a final extension at 72°C for 10 minutes. Amplicons obtained were sequenced using ABI Prism Sequencing kit (BigDye Terminator Cycle) at SciGenom, India. 

### 2.6. Data Analysis

The homologue searching of the nucleotide sequence was performed with the Basic Local Alignment Search Tool (BLAST) through NCBI server (http://www.ncbi.nlm.nih.gov/blast). The nucleotide sequence was translated into amino acid sequence by the DNA-Protein translation tool provided by Expert Protein Analysis System, ExPASy (http://au.expasy.org/). Phylogenetic tree was constructed by the neighbour-joining (NJ) method and maximum likelihood (ML) method based on amino acid sequence of histone H2A and nucleotide sequence of cytochrome oxidase subunit I, using MEGA version 5.05. Confidence in estimated relationships of ML and NJ tree topologies was evaluated by a bootstrap analysis with 100 and 1,000 replicates with MEGA version 5.0. Kimura 2 parameter (K2P) model was used to construct NJ and ML tree for CO1 genes. The cleavage sites of proteolytic enzymes on the deduced amino acid sequence were predicted using PeptideCutter Tool (http://web.expasy.org/peptide_cutter/). Molecular weight, isoelectric point, and stability of each peptide sequence were calculated using ProtParam software (http://web.expasy.org/protparam/). The primary structure of deduced amino acid sequences was compared with previously reported histone H2A derived AMPs from other species by using the multiple sequence alignment program CLUSTALW. Charge over a range of pH and concentration of peptides was calculated using Protein Calculator v 3.3 (http://www.scripps.edu/~cdputnam/protcalc.html) and hydrophobicity using PepDraw tool (http://www.tulane.edu/~biochem/WW/PepDraw/index.html). Three-dimensional arrangement of peptide was created in PyMOL software using data generated by SWISS-MODEL [18–20]. 

## 3. Results

**Figure 1:**
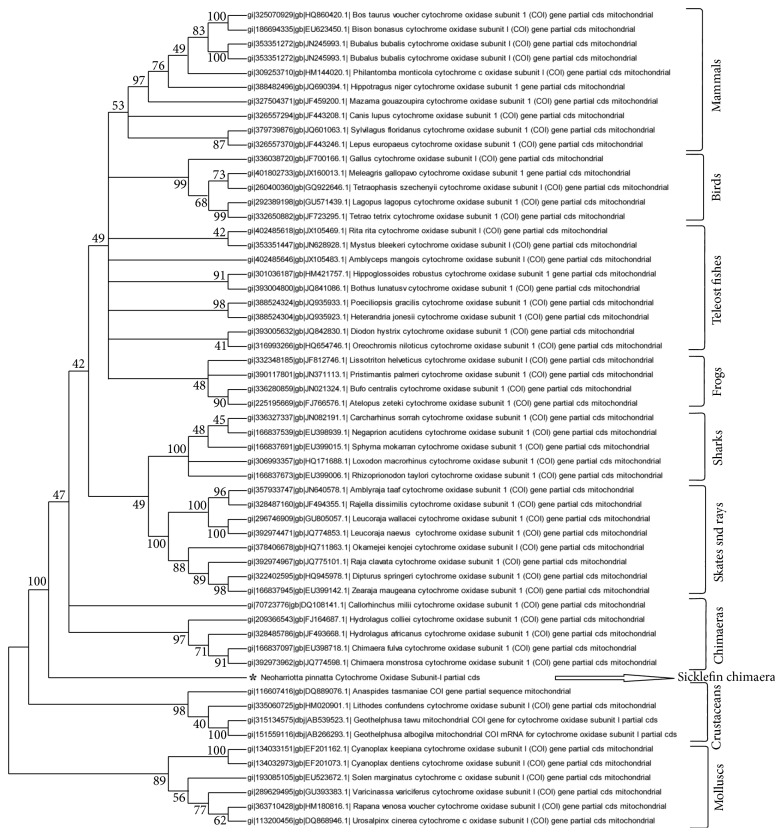
A bootstrapped neighbor-joining tree obtained using MEGA version 5.05 illustrating the phylogenetic relationship of *N. pinnata* based on the nucleotide sequence of cytochrome oxidase subunit-1 gene.

The CO1 primers amplified a 710 bp region of the gene mitochondrial cytochrome oxidase subunit I (GenBank ID JX297203). BLAST analysis of nucleotide sequences confirmed the identity of the organism as *N. pinnata* showing 99% similarity to GenBank ID: HM239670.1 *Neoharriotta pinnata*. Phylogenetic relationship of *N. pinnata* to other organisms was established based on the nucleotide sequence comparisons of CO1. Phylogenetic relationship of *N. pinnata* to other organisms was virtually identical in both NJ tree and ML tree. NJ tree represented in Figure 1 gets broadly divided into six clusters. Cluster one includes mammals, cluster two includes three subclusters representing birds, teleost fishes, and frogs, cluster three includes two subclusters of cartilaginous fishes, one representing sharks and the other representing skates and rays, cluster four includes fishes belonging to Holocephali group, cluster five represents crustaceans, and cluster six includes molluscs. *N. pinnata* though closely related to Holocephalan fishes occupies a position in-between the vertebrate and invertebrate groups.

**Figure 2:**
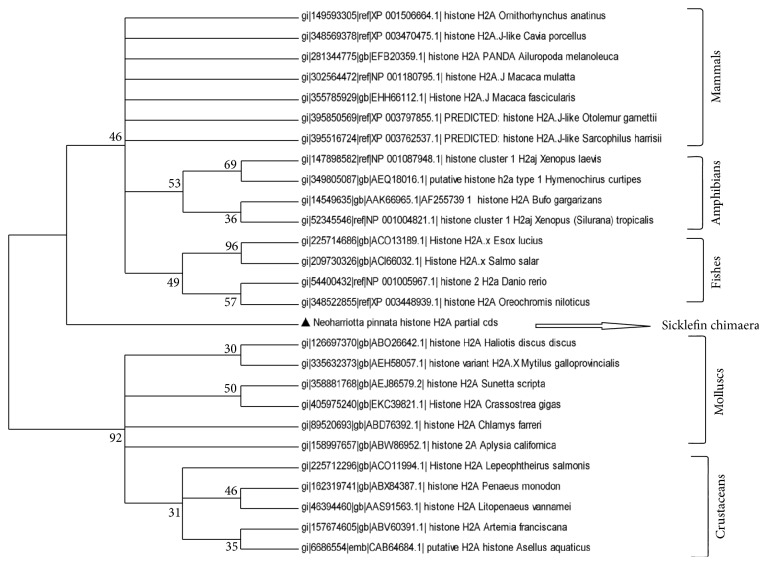
A bootstrapped neighbor-joining tree obtained using MEGA version 5.05 illustrating relationships between the amino acid sequence of histone H2A of* N. pinnatta* to the amino acid sequences of previously reported histone H2A from different organisms.

**Figure 3:**
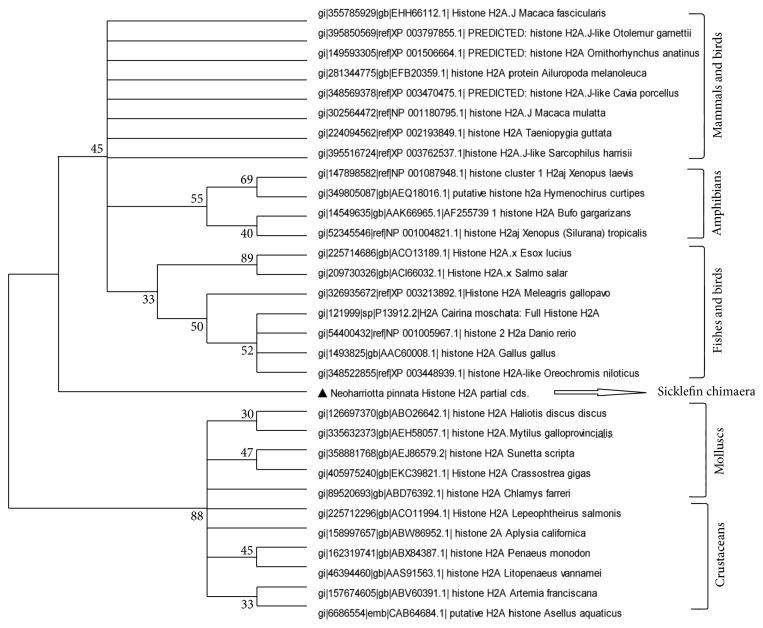
A bootstrapped neighbor-joining tree obtained using MEGA version 5.05 illustrating relationships between the amino acid sequence of histone H2A of* N. pinnatta* to the amino acid sequences of previously reported histone H2A from invertebrates, fishes, amphibians, birds, and mammals. Birds can be seen aligned with mammals and fishes.

RT-PCR amplification of the mRNA from Sicklefin Chimaera yielded a 243 bp fragment cDNA encoding 81 amino acid residues. The obtained nucleotide and deduced amino acid sequences were deposited in GenBank database (GenBank ID: JX297204). BLAST analysis of the nucleotide and deduced amino acid sequences revealed that the peptides belonged to histone H2A family. Bootstrap distance tree calculated using deduced amino acid sequence confirmed its similarity with previously reported histone H2A sequences deposited in GenBank database. Bootstrap distance tree was calculated using NJ method and ML method. Phylogenetic relationship of histone H2A of *N. pinnata *to histone H2A of other organisms was found to be virtually identical in both NJ tree and ML tree. The phylogenetic distance tree based on amino acid sequence of histone H2A is represented in Figure 2. The phylogenetic tree gets divided into two main clusters. Cluster one denotes histone H2A sequences of vertebrates, and cluster two represents that of invertebrates. The vertebrate group could be classified into three subclusters representing mammals, amphibians, and fishes, while invertebrate group could be classified in mollusc and crustacean sub-clusters. Birds, when included for construction of phylogenetic tree, grouped with both mammals and fishes (Figure 3). In case of histone H2A also, *N. pinnata* was found to occupy a position in between the vertebrate and invertebrate clusters, though more closely related to vertebrates than invertebrates. Histone H2A protein of *N. pinnatta* differs from other reported histone H2A proteins in having amino acid His at position 34 and Asp at position 42 from the N-terminus. In all other previously reported sequence of histone H2A, Lys and Glu are present at the corresponding position of His and Asp. Histone H2A protein of *N. pinnatta *has Val at position 31 and Thr at position 60 from the N-terminus. Histone H2A reported in vertebrates is similar to *N. pinnatta* in having Val and Thr at corresponding positions, but histone H2A reported from invertebrates has Ile and Ala at corresponding positions. *N. pinnatta* has Val at position 63, a feature found in invertebrates, as all histone H2A reported from them have Val at corresponding position. The scenario is different in case of all other vertebrates as they have Ile at corresponding position. Histone H2A of *N. pinnatta* displays similarities and dissimilarities with both vertebrates and invertebrates and thereby occupies a position in-between the two (for details see supplementary Figure in Supplementary Material available online at http://dx.doi.org/10.1155/2013/930216).

**Figure 4:**
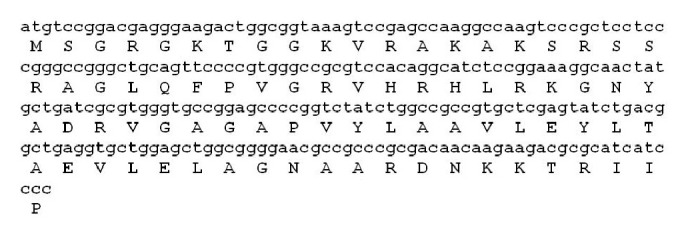
Nucleotide and amino acid sequence of histone H2A protein from *N. pinnatta*.

**Figure 5:**
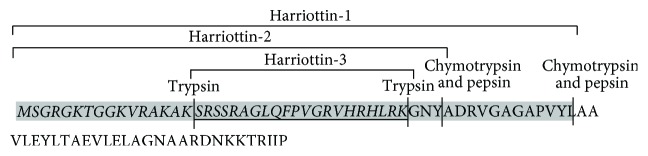
Potential cleavage site of proteolytic enzymes resulting in formation of Harriottin-1, -2, and -3 is demonstrated.

**Figure 6:**
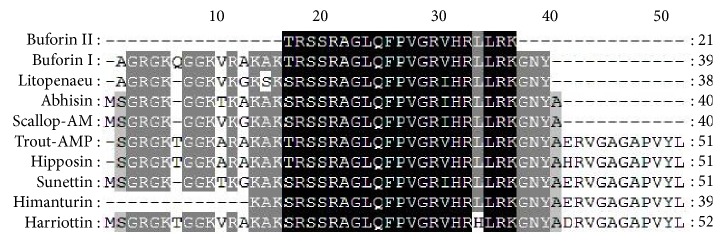
ClustalW multiple alignment of Harriottin-1 (*N. pinnatta*) with Buforin I and II (*Bufo bufo gargarizans*), Hipposin (*Hippoglossus hippoglossus*), Rainbow Trout H2A (*Oncorhynchus mykiss*), Litopenaeus AMP (*Litopenaeus vannamei*), Scallop AMP (*Chlamys farreri*), Abhisin (*Haliotis discus*), Sunettin (*Sunetta scripta*), and Himanturin (*Himantura pastinacoides*).

**Figure 7:**
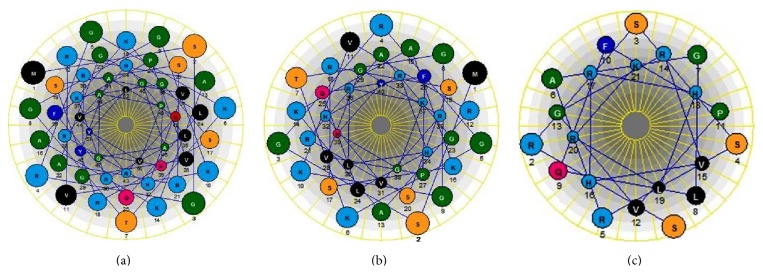
Schiffer-Edmundson helical wheel diagram demonstrating probable amphipathic *α*-helical conformation of Harriottin-1 (a), Harriottin-2 (b), and Harriottin-3 (c).

**Figure 8:**
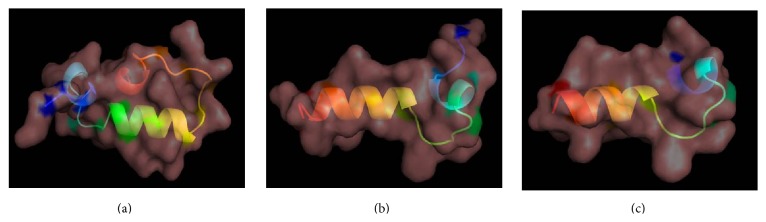
Predicted 3-dimentional structural arrangements of Harriottin-1 (a), Harriottin-2 (b), and Harriottin-3 (c) generated using PyMol software.

The nucleotide sequence and the deduced amino acid sequence of histone H2A amplified from sicklefin chimaera are presented in Figure 4. Analysis of functional aspects and chemical properties of the histone H2A protein were carried out using reliable computer based programs. The PeptideCutter tool predicts proteolytic enzymes, chymotrypsin, and pepsin to have a potential cleavage site at position 52 and 40 from N-terminus of histone H2A of sicklefin chimaera. Cleaving the protein at position 52 would release Harriottin-1, a peptide sharing similarity with Hipposin. Proteolytic activity of these enzymes at position 40 would result in the release of a peptide termed as Harriottin-2 which is similar to Buforin I reported from toad. Enzyme trypsin was found to have potential cleavage sites at position 16 and 37 from the N-terminus. Trypsin mediated processing of *N. pinnata *histone H2A would result in the formation of a 21 mer peptide, Harriottin-3 having a sequence resembling Buforin II. Diagrammatic representation of the cleavage site of enzymes and release of the three Harriottins is presented in Figure 5. Sequence analysis of the peptides was carried out using ProtParam software which predicted Harriottin-1, -2, and -3 to have molecular weights of 5.56 kDa, 4.39 kDa, and 2.44 kDa, respectively, and a theoretical isoelectric point (pI) of 12.01, 12.41, and 12.60, respectively. ProtParam software estimated the half-life of Harriottin-1, -2, and -3 to be more than 20 hour in yeast and more than 10 hours in *E. coli*. In case of mammalian reticulocytes Harriottin-1 and -2 were estimated to have a half-life of 30 hours, whereas Harriottin-3 was found to have a half-life of 1.9 hours. Multiple-sequence alignment of the amino acid sequences of Harriottin-1 with previously reported histone H2A derived AMPs revealed that the peptide showed similarity to previously reported histone H2A derived AMPs like Buforin I, Buforin II, Hipposin, Himanturin, Abhisin, Sunettin, and histone H2A derived AMPs reported from* Oncorhynchus mykiss*, *Litopenaeus vannamei,* and *Chlamys farreri *(Figure 6). Only Harriottin-1 was considered for multiple-sequence alignment as it covers sequences of both Harriottin-2 and Harriottin-3. All three Harriottins were found to have an overall net positive charge. At pH 4, 7, and 10, Harriottin-1 was found to have a charge of 14.8, 12.4, and 7.4; Harriottin-2 had a net positive charge of 14.1, 12.4, and 7.9, while Harriottin-3 displayed a net positive charge of 7.1, 5.4, and 4. Hydrophobicity of Harriottin-1, -2, and -3 were found to be +52.17 kcal/mol (30%), +45.66 kcal/mol (25%), and +24.37 kcal/mol (28%) as predicted by PepDraw. Analysis of Harriottins using Protean module of the DNASTAR Lasergene sequence analysis software suite revealed that Harriottin-1 and -2 will have a concentration of 1.87 mg/mL and 2.95 mg/mL for an absorbance of 1 OD measured at 280 nm, whereas Harriottin-3 will not give any reading at 280 nm wavelength, as it lacks Thr, Cys, and Trp. The module further predicts that 1 *μ*g of the Harriottin-1, -2, and -3 would contain 179.75 pmoles, 227.63 pmoles, and 409.03 pmoles, respectively. Schiffer-Edmundson helical wheel modeling of the peptides using Protean module revealed clustering of hydrophobic and hydrophilic/basic residues on opposing sides of the helical wheel (Figure 7). This result suggests an amphipathic nature and an *α*-helical structure for the Harriottins. Three-dimensional arrangement of Harriottins is shown in Figure 8. Analysis of Harriottins for their antimicrobial activity was carried out with Antimicrobial Peptide Predictor Program (http://aps.unmc.edu/AP/main.php) which predicts them to be antimicrobial peptides with a protein binding potential of 2.58 kcal/mol, 3.26 kcal/mol, and 3.83 kcal/mol for Harriottin-1, -2, and -3, respectively.

## 4. Discussion

The present study describes characterization of Harriottin-1, -2, and -3, with 52, 40, and 21 amino acid peptides identified from histone H2A of *N. pinnata* and phylogenetic analysis of the organism based on CO1 and histone H2A. One of the commonly used molecular markers for taxonomic identification of a species is the CO1 gene, comparison of which provides a reliable determination of the phylogenetic relationship of a species indicating its position in the evolutionary tree. The phylogenetic relationship of *N. pinnatta* based on nucleotide sequence of CO1 gene was analyzed by NJ method and ML method. As expected *N. pinnatta* was found to be closely related to Holocephalan fishes but was found to occupy a position between vertebrates and invertebrates. Order Chimaeriformes to which chimaeras belong include three families: Chimaeridae, Callorhinchidae, and Rhinochimaeridae. Results of the phylogenetic analysis based on CO1 gene indicate that Rhinochimaeridae represented by* N. pinnatta* appears to be more primitive of the three.

The phylogenetic relationship of histone H2A amino acid sequence of *N. pinnatta* to the amino acid sequence of previously reported histone H2A proteins from various organisms was carried out using NJ method and ML method. The molecular phylogenetic tree based on amino acid sequences of previously reported histone H2A derived AMPs demonstrates that the members of the family are derived from a common ancestor by a series of evolutionary changes. Selected histone H2A derived antimicrobial peptide sequences got divided into two major groups, that is, vertebrates and invertebrates. The boot strap distance tree calculated reveals that histone H2A protein of *N. pinnatta* can align with the vertebrate group, but the lineage is distant enough to conclude that it occupies a position between vertebrate and invertebrate groups. Birds when included in the phylogenetic tree did not form a group of their own and was found to align with fishes and mammals. Evolution of histone H2A is not clearly demarcated in birds and this indicates that the histone H2A has a highly conserved sequence. Even though the rate of evolution is slow in histone H2A, well-marked differences can be observed in case of *N. pinnata*. Histone H2A protein of *N. pinnatta *diverges from other reported histone H2A proteins at amino acid position 34 and 42 from the N-terminus where it has His and Asp, respectively. Lys and Glu is present at the corresponding position of His and Asp in all other reported sequences of histone H2A. Since Lys and His as well as Asp and Glu belong to same characteristic group in amino acid classification, their interchange would not have much effect on the property of the protein. The region of histone H2A corresponding to amino acid position 16 to 51 in histone H2A of *N. pinnatta* is a highly conserved region in animal kingdom. *N. pinnatta* differs from histone H2A of other organisms in this region at two positions (position 34 and 42) which clearly indicates that sicklefin chimaera has followed a different path of evolution. *N. pinnatta* represents Holocephalan fishes which are believed to be branched off from their sister group of sharks and rays and have remained isolated ever since. This is quite evident from the results of phylogenetic analysis based on histone H2A amino acid sequence of *N. pinnatta* which shows similarity to both vertebrates and invertebrates and at the same time differs from both of them. Since histone H2A sequences of cartilaginous fishes are scarce in GenBank database a detailed investigation was not possible. Histone genes represent much conserved regions, and therefore evolutionary analyses of histones should provide important information with regard to the phylogenetic relationships of distant/closely related organisms.

Harriottins exhibited high sequence similarity with previously reported histone H2A derived AMPs. Harriottins are highly cationic peptides with amphipathic nature and *α*-helical structure, characteristic to all histone H2A derived AMPs. Harriottin-1, -2, and -3 were found to be rich in arginine (15, 18, and 23%), glycine (17, 18, and 10%), serine (8, 10, and 14%), valine (10, 8, and 10%), and alanine (11, 8, and 5%) as reported in all other histone H2A derived AMPs. All histone H2A derived AMPs reported to date from various sources are derived from N-terminal region of histone H2A, thereby suggesting its importance in innate immune response of an organism. Histone H2A fragments with antimicrobial activity reported from vertebrates and invertebrates clearly indicate the role of histone H2A as a potential precursor for highly potent antimicrobial peptides. In Asian Toad *Bufo bufo gargarizans*, the intact histone H2A protein is secreted into the stomach, and Buforin I is produced by the action of pepsin isozymes cleaving the Try 39-Ala 40 bond of intact protein [21]. Similarly in Cat Fish (*Parasilurus asotus*), parasin I is produced by cleavage of Ser19-Arg20 bond of histone H2A by cathepsin D found in skin mucus of the fish [22]. PeptideCutter tool predicts proteolytic enzymes trypsin, chymotrypsin, and pepsin to have potential cleavage sites in the histone H2A of sicklefin chimaera which presented the possibility of formation of three fragments similar to previously reported histone H2A derived AMPs. A 52-mer fraction similar to hipposin was termed as Harriottin-1; a 40-mer fraction resembling Buforin I was termed as Harriottin-2, and a third 21-mer fraction comparable to Buforin II was given the name Harriottin-3.

Histone H2A derived antimicrobial peptides are known to exhibit broad spectrum activity against bacteria and fungi. Hipposin and Buforins are the most studied histone H2A derived antimicrobial peptides. Hipposin exhibited strong antibacterial activity against several Gram-positive and Gram-negative bacteria, and activity could be detected down to a concentration of 1.6 *μ*g/mL [10]. Harriottin-1 has a sequence and structure similar to Hipposin and therefore would have a similar activity. Buforins are among one of the most potent antimicrobial peptides. In addition to their broad spectrum activity against bacteria and fungi [23], they also possess antiendotoxic and anticancer activities [24]. Harriottin-2 and -3 would be expected to match the activity of Buforin I and II, respectively, by virtue of their sequence and structure. Buforin II does not cause significant membrane permeabilization [25] but brings about the lysis of bacterial cells by readily entering the cells in vivo and by interacting with intracellular nucleic acids [26, 27]. NMR structural studies showed that proline at position 11 serves as a hinge between a C-terminus helix and an N-terminal region with an extended helical structure [28]. This sole proline residue (Pro_11_) of Buforin II is necessary for effective translocation across cell membrane [25, 26]. Presence of proline at position 11 and the resulting proline hinge as in Buforin II was also detected to be a characteristic feature of Harriottin-3. Presence of proline hinge seems to indicate that the antimicrobial activity of Harriottin-3 lies in its ability to interact with nucleic acid rather than membrane permeabilization. Antimicrobial peptides are also viewed as agents with therapeutic potential against cancer cells [29]. Buforin II exhibits selective cytotoxicity against cancer cells through interaction with cell surface gangliosides, and once inside the cell they induce mitochondria-dependent apoptosis [30]. Buforin II does not exhibit cytotoxic activity of any kind against normal mammalian cells [31]. Having a structure similar to Buforin II makes Harriottin-3 potential candidates for anticancer research. Antimicrobial Peptide Predictor Program (http://aps.unmc.edu/AP/main.php) predicted Harriottins to be AMPs, since Harriottin-1, -2, and -3 form alpha helices and possess 6, 4, and 4 residues, respectively, on the same hydrophobic surface which assist them to interact with membranes. Harriottin-1, -2, and -3 illustrate all the characteristic features of AMPs including high cationicity, higher hydrophobic residue, and elevated protein binding potential, that is, 2.58, 3.26, and 3.83 kcal/mol, respectively.

## 5. Conclusion

Three peptides containing antimicrobial sequence motif from the histone H2A of *N. pinnatta* were identified and named as Harriottin-1, -2, and -3. High sequence similarity of Harriottin-1, 2 and 3 to previously reported potent histone H2A derived AMPs and their similarity to traditional antimicrobial peptides in physicochemical properties strongly endorse Harriottins to be considered as peptides with antimicrobial activity. The study was taken up as an initiator to investigate the role of histone derived AMPs in Holocephalan fishes, and more research in this area would reveal new facets of innate immunity in this less understood group of fishes. The study gives a comparative account of CO1 and H2A nucleotide sequences in the molecular taxonomic identification of members of the animal kingdom. Birds get grouped both with fishes and mammals, but not with amphibians, which is really intriguing. The study also offers an insight into the evolutionary divergence of *N. pinnatta *with respect to CO1 gene and histone H2A occupying an intermediate position with respect to invertebrates and invertebrates.

## Supplementary Material

Multiple alignment of amino acid sequence of histone H2A of N. pinnatta to the amino acid sequence of histone H2A previously reported from various organisms demonstrates the differences in N. pinnatta to other reported histone H2A proteins. Histone H2A of N. pinnatta is peculiar in having His at position 34 and Asp at position 42 from the N-terminus while in all other previously reported sequence of histone H2A, presence of Lys and Glu can be seen at the corresponding positions . Val at position 31 and Thr at position 60 in histone H2A of N. pinnatta. has been replaced by Val and Thr in vertebrates, whereas, histone H2A reported from invertebrates have Ile and Ala at corresponding positions. N. pinnatta has Val at position 63, a feature it has in common with invertebrates. Vertebrates have Ile at corresponding position.

## References

[1] Bulet P., Hetru C., Dimarcq J., Hoffmann D. (1999). Antimicrobial peptides in insects; structure and function. *Developmental and Comparative Immunology*.

[2] Jiang Z., Vasil A. I., Hale J. D., Hancock R. E. W., Vasil M. L., Hodges R. S. (2008). Effects of net charge and the number of positively charged residues on the biological activity of amphipathic *α*-helical cationic antimicrobial peptides. *Biopolymers*.

[3] Ellis A. E. (1974). Non-specific defense mechanisms in fish and their role in disease processes. *Developments in Biological Standardization*.

[4] Manning M. J., Blanck K. D., Pickering A. D. (1998). Immune defence systems. *Biology of Farmed Fish*.

[5] Bly J. E., Clem L. W. (1991). Temperature-mediated processes in teleost immunity: in vitro immunosuppression induced by in vivo low temperature in channel catfish. *Veterinary Immunology and Immunopathology*.

[6] Kawasaki H., Iwamuro S. (2008). Potential roles of histones in host defense as antimicrobial agents. *Infectious Disorders*.

[7] Li C., Song L., Zhao J. (2007). Preliminary study on a potential antibacterial peptide derived from histone H2A in hemocytes of scallop *Chlamys farreri*. *Fish and Shellfish Immunology*.

[8] Park I. Y., Park C. B., Kim M. S., Kim S. C. (1998). Parasin I, an antimicrobial peptide derived from histone H2A in the catfish, *Parasilurus asotus*. *FEBS Letters*.

[9] Richards R. C., O'Neil D. B., Thibault P., Ewart K. V. (2001). Histone H1: an antimicrobial protein of Atlantic salmon (*Salmo salar*). *Biochemical and Biophysical Research Communications*.

[10] Birkemo G. A., Lüders T., Andersen Ø., Nes I. F., Meyer J. N. (2003). Hipposin, a histone-derived antimicrobial peptide in Atlantic halibut (*Hippoglossus hippoglossus* L.). *Biochimica et Biophysica Acta*.

[11] Fernandes J. M. O., Kemp G. D., Molle G. M., Smith V. J. (2002). Anti-microbial properties of histone H2A from skin secretions of rainbow trout, *Oncorhynchus mykiss*. *Biochemical Journal*.

[12] Sathyan N., Philip R., Chaithanya E. R., Kumar P. R. A., Antony S. P. (2012). Identification of a histone derived, putative antimicrobial peptide Himanturin from round whip ray *Himantura pastinacoides* and its phylogenetic significance. *Results in Immunology*.

[13] Chaithanya E. R., Philip R., Sathyan N., Kumar P. R. A. (2013). Molecular characterization and phylogenetic analysis of a histone-derived antimicrobial peptide teleostin from the marine teleost fishes, *Tachysurus jella* and *Cynoglossus semifasciatus*. *ISRN Molecular Biology*.

[14] Patat S. A., Carnegie R. B., Kingsbury C. (2004). Antimicrobial activity of histones from hemocytes of the Pacific white shrimp. *European Journal of Biochemistry*.

[15] Sathyan N., Philip R., Chaithanya E. R., Kumar P. R. A., Antony S. P., Singh I. S. B. (2012). Identification of a histone derived, putative antimicrobial peptide sunettin from marine clam *Sunetta scripta*. *Blue Biotechnology Journal*.

[16] Sathyan N., Philip R., Chaithanya E. R., Kumar P. R. A. (2012). Identification and molecular characterization of molluskin, a histone-H2A-derived antimicrobial peptide from molluscs. *ISRN Molecular Biology*.

[17] Folmer O., Black M., Hoeh W., Lutz R., Vrijenhoek R. (1994). DNA primers for amplification of mitochondrial cytochrome c oxidase subunit I from diverse metazoan invertebrates. *Molecular Marine Biology and Biotechnology*.

[18] Guex N., Peitsch M. C. (1997). SWISS-MODEL and the Swiss-PdbViewer: an environment for comparative protein modeling. *Electrophoresis*.

[19] Schwede T., Kopp J., Guex N., Peitsch M. C. (2003). SWISS-MODEL: an automated protein homology-modeling server. *Nucleic Acids Research*.

[20] Arnold K., Bordoli L., Kopp J., Schwede T. (2006). The SWISS-MODEL workspace: a web-based environment for protein structure homology modelling. *Bioinformatics*.

[21] Kim H. S., Yoon H., Minn I. (2000). Pepsin-mediated processing of the cytoplasmic histone H2A to strong antimicrobial peptide buforin I. *The Journal of Immunology*.

[22] Cho J. H., Park I. Y., Kim H. S., Lee W. T., Kim M. S., Kim S. C. (2002). Cathepsin D produces antimicrobial peptide parasin I from histone H2A in the skin mucosa of fish. *The FASEB Journal*.

[23] Park C. B., Kim M. S., Kim S. C. (1996). A novel antimicrobial peptide from *Bufo bufo gargarizans*. *Biochemical and Biophysical Research Communications*.

[24] Cho J. H., Sung B. H., Kim S. C. (2009). Buforins: histone H2A-derived antimicrobial peptides from toad stomach. *Biochimica et Biophysica Acta*.

[25] Kobayashi S., Takeshima K., Park C. B., Kim S. C., Matsuzaki K. (2000). Interactions of the novel anfimicrobial peptide buforin 2 with lipid bilayers: proline as a translocation promoting factor. *Biochemistry*.

[26] Park C. B., Yi K. S., Matsuzaki K., Kim M. S., Kim S. C. (2000). Structure-activity analysis of buforin II, a histone H2A-derived antimicrobial peptide: the proline hinge is responsible for the cell-penetrating ability of buforin II. *Proceedings of the National Academy of Sciences of the United States of America*.

[27] Uyterhoeven E. T., Butler C. H., Ko D., Elmore D. E. (2008). Investigating the nucleic acid interactions and antimicrobial mechanism of buforin II. *FEBS Letters*.

[28] Yi G. S., Park C. B., Kim S. C., Cheong C. (1996). Solution structure of an antimicrobial peptide buforin II. *FEBS Letters*.

[29] Hoskin D. W., Ramamoorthy A. (2008). Studies on anticancer activities of antimicrobial peptides. *Biochimica et Biophysica Acta*.

[30] Lee H. S., Park C. B., Kim J. M. (2008). Mechanism of anticancer activity of buforin IIb, a histone H2A-derived peptide. *Cancer Letters*.

[31] Takeshima K., Chikushi A., Lee K. K., Yonehara S., Matsuzaki K. (2003). Translocation of analogues of the antimicrobial peptides magainin and buforin across human cell membranes. *The Journal of Biological Chemistry*.

