# Supplementary figures and images for: Characterization of Histone H2A Derived Antimicrobial Peptides, Harriottins, from Sicklefin Chimaera Neoharriotta pinnata (Schnakenbeck, 1931) and Its Evolutionary Divergence with respect to CO1 and Histone H2A
Source: ISRN Mol Biol. 2013 Jun 2;2013:930216. doi: 10.1155/2013/930216 (PMC4890863; doi:10.1155/2013/930216)

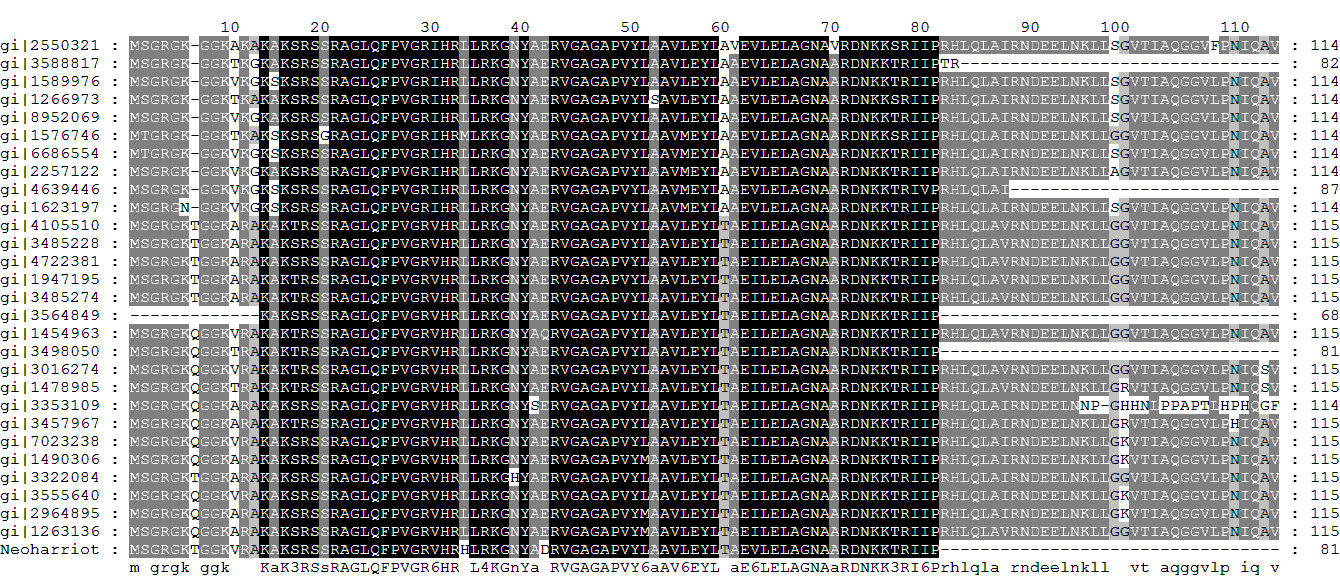

Supplement: Supplementary file 1 — Multiple alignment of amino acid sequence of histone H2A of N. pinnatta to the amino acid sequence of histone H2A previously reported from various organisms demonstrates the differences in N. pinnatta to other reported histone H2A proteins. Histone H2A of N. pinnatta is peculiar in having His at position 34 and Asp at position 42 from the N-terminus while in all other previously reported sequence of histone H2A, presence of Lys and Glu can be seen at the corresponding positions . Val at position 31 and Thr at position 60 in histone H2A of N. pinnatta. has been replaced by Val and Thr in vertebrates, whereas, histone H2A reported from invertebrates have Ile and Ala at corresponding positions. N. pinnatta has Val at position 63, a feature it has in common with invertebrates. Vertebrates have Ile at corresponding position. [file 930216.f1.png]
